# Inferior parietal lobule and early visual areas support elicitation of individualized meanings during narrative listening

**DOI:** 10.1002/brb3.1288

**Published:** 2019-04-11

**Authors:** Satu Saalasti, Jussi Alho, Moshe Bar, Enrico Glerean, Timo Honkela, Minna Kauppila, Mikko Sams, Iiro P. Jääskeläinen

**Affiliations:** ^1^ Department of Psychology and Logopedics, Medical Faculty University of Helsinki Helsinki Finland; ^2^ Brain and Mind Laboratory, Department of Neuroscience and Biomedical Engineering Aalto University School of Science Espoo Finland; ^3^ Advanced Magnetic Imaging (AMI) Centre Aalto NeuroImaging, School of Science Aalto University Espoo Finland; ^4^ Gonda Multidisciplinary Brain Research Center Bar‐Ilan University Ramat Gan Israel; ^5^ Helsinki Institute of Information Technology, Aalto University Espoo Finland; ^6^ Department of Digital Humanities University of Helsinki Helsinki Finland; ^7^ Department of Computer Science Aalto University School of Science Espoo Finland

**Keywords:** brain mechanisms, functional magnetic resonance imaging, interindividual differences, meaning, semantics, similarity

## Abstract

**Introduction:**

When listening to a narrative, the verbal expressions translate into meanings and flow of mental imagery. However, the same narrative can be heard quite differently based on differences in listeners' previous experiences and knowledge. We capitalized on such differences to disclose brain regions that support transformation of narrative into individualized propositional meanings and associated mental imagery by analyzing brain activity associated with behaviorally assessed individual meanings elicited by a narrative.

**Methods:**

Sixteen right‐handed female subjects were instructed to list words that best described what had come to their minds while listening to an eight‐minute narrative during functional magnetic resonance imaging (fMRI). The fMRI data were analyzed by calculating voxel‐wise intersubject correlation (ISC) values. We used latent semantic analysis (LSA) enhanced with Wordnet knowledge to measure semantic similarity of the produced words between subjects. Finally, we predicted the ISC with the semantic similarity using representational similarity analysis.

**Results:**

We found that semantic similarity in these word listings between subjects, estimated using LSA combined with WordNet knowledge, predicting similarities in brain hemodynamic activity. Subject pairs whose individual semantics were similar also exhibited similar brain activity in the bilateral supramarginal and angular gyrus of the inferior parietal lobe, and in the occipital pole.

**Conclusions:**

Our results demonstrate, using a novel method to measure interindividual differences in semantics, brain mechanisms giving rise to semantics and associated imagery during narrative listening. During listening to a captivating narrative, the inferior parietal lobe and early visual cortical areas seem, thus, to support elicitation of individual meanings and flow of mental imagery.

## 1 INTRODUCTION

When listening to a narrative, the verbal expressions translate into propositional meanings (i.e., semantics) along with the associated mental imagery, with the keen listener seeing with his/her “mind's eye” the objects, environments, actions, and events in the story. The intriguing question of how the human brain codes the semantics of language has been under investigation for decades (Binder, Desai, Graves, & Conant, 2009). Brain areas sensitive to word meanings have been observed in the temporal, parietal, and frontal cortices (Binder & Desai, 2011; Binder et al., 2009). It has been suggested that inferior parietal regions act as convergence zones for concept and event knowledge, receiving input from sensory, action, and emotion systems (Binder et al., 2009). Recently, in a study where word‐meaning categories occurring in a narrative were mapped onto human cerebral cortex using functional magnetic resonance imaging (fMRI) (Huth, Heer, Griffiths, Theunissen, & Jack, 2016), the results both agreed with previous meta‐analysis of semantic areas of the human brain (Binder et al., 2009) and extended our understanding, as they disclosed how semantic categories tile the cortical surface. The semantic representations were not confined to left hemisphere, but were observed predominantly bilaterally (Huth et al., 2016). However, information is represented in human brain in multiple ways (Pearson & Kosslyn, 2015), and listening to a captivating story may, in addition to linguistic semantics, also activate processes related to mental imagery as one sees events with the “mind's eye” (Sadoski, 1983; Sadoski, Goetz, Olivarez, Lee, & Roberts, 1990). Previous empirical evidence suggests that when a person forms mental imagery, visual cortical areas are activated, which are also the first cortical areas to receive real visual signal from the eyes (Kosslyn, Ganis, Thompson, & Hall, 2001; Pearson & Kosslyn, 2015), though there are differences between individuals in the strength of visual imagery (Bergmann, Genç, Kohler, Singer, & Pearson, 2016).

What previous studies have not yet addressed is that stories can be experienced quite differently (Jääskeläinen, Pajula, Tohka, Lee, & Kuo, 2016) based on differences in previous experiences (Cabeza & Jacques, 2007), for example, upon hearing the word “dog” one person can come to think of a happy Collie, another an angry Rottweiler. Given such interindividual differences, we hypothesized that by analyzing brain activity based on behaviorally assessed individual semantics (Bar, 2007) elicited by a narrative we can disclose brain regions supporting the elicitation of individual semantics and mental imagery during story listening**.** For a recent similar type of approach, see (Nguyen, Vanderwal, & Hasson, 2019). We presented an eight‐minute narrative describing daily events in a woman's life to 16 healthy females during 3T‐fMRI, and afterwards asked subjects to report, by listing descriptive words, what had come to their minds while listening to the narrative during fMRI. We then utilized latent semantic analysis (LSA; Landauer & Dutnais, 1997) combined with WordNet (Liu, Wang, Buckley, & Zhou, 2011; Miller, 1995) to quantify similarities/differences in these word listings between each pair of subjects and tested, and using representational similarity analysis (RSA; Kriegeskorte, Mur, & Bandettini, 2008), whether similarities/differences in the individualized meanings predicted similarities/differences in brain activity as quantified using intersubject correlations (Hasson, Nir, Levy, Fuhrmann, & Malach, 2004; Kauppi, Jääskeläinen, Sams, & Tohka, 2010). We specifically hypothesized to see involvement of brain areas such as the inferior parietal lobe and visual cortical areas identified in previous studies as core semantic processing areas (Binder et al., 2009) and areas activated during mental imagery (Pearson & Kosslyn, 2015). Furthermore, by demonstrating how interindividual differences in semantic representations can be measured and utilized to map the semantic areas in the brain, our findings also provide an important methodological extension for studying the human semantic system.

## 2 MATERIALS AND METHODS

### 2.1 Participants

Sixteen healthy, right‐handed (Edinburgh handedness inventory (Oldfield, 1971)) female volunteers (ages 20–42) participated in the study. Subjects reported normal hearing and normal or corrected to normal (with contact lenses) vision, and had no psychiatric or neurological disabilities. All subjects gave an informed consent prior to their inclusion in the study, and received monetary compensation for their time (2.5 hr) used for taking part of the experiment. The study was approved by the research ethics committee of Aalto University and it was conducted in accordance with the Helsinki Declaration for Human studies.

### 2.2 Stimuli and experimental design

The behavioral and fMRI data for the current experiment were obtained in parallel with a broader‐scope fMRI experiment (*N* = 29) investigating brain mechanisms during listening (audio‐only), reading (time‐locked text‐only), and lipreading (silent video) a narrative (Saalasti et al., 2018), as well as an unintelligible, gibberish version of the each of the intact narrative condition. Duration of the narrative was 7 min 54 s. The narrative described, from first‐person perspective, daily events in a life of a woman (for original Finnish and English‐translated versions of the story, see Appendices A and B below). The gibberish was created by replacing speech sounds from each word of the original narrative, but keeping the suffixes that indicated syntax unchanged. This resulted in meaningless string of speech sounds that had very similar acoustic properties and structure (syntax) than the original narrative, but no content (semantics). Results related to the gibberish narrative will be reported separately. The stimulus sequence in the full experimental design consisted of the narrative presented six times, that is, three intact (lipread, read, and listened), and three gibberish (lipread, read, and listened) versions of the same narrative. In the broader‐scope experiment, presentation order of the conditions (gibberish and intact lipread, read, and listened) was counterbalanced to avoid order effects. Because comprehension of the lipread narrative was limited, the word–list associations were obtained only from a subset of subjects who listened or read the narrative first, resulting in 16 subjects reported in the current study. Eleven of the subjects heard the narrative as naïve in the scanner, while five of them heard the narrative after the reading condition.

Presentation software (Neurobehavioral Systems Inc., Albany, California, USA) was used for presenting the stimuli. The audio stimuli were played with an MRI‐compatible in‐ear earbuds (Sensimetrics S14 insert earphones). In addition, MRI‐safe protecting earmuffs were placed over the earbuds for noise removal and safety. Sound intensity was adjusted for each subject during a dummy echo‐planar imaging (EPI) sequence before the actual experiment to be loud enough to be heard over the scanner noise by playing example stimuli that were normalized to the same level as the auditory stories. In the MRI scanner, the stimulus videos and texts were back‐projected on a semitransparent screen, using a Panasonic PT‐DZ110XEJ projector (Panasonic Corporation, Osaka, Japan). The viewing distance was 35 cm.

During narrative presentation, the subjects' brain hemodynamic activity was recorded with fMRI (Siemens 3‐Tesla Skyra, Erlangen, Germany; standard 20‐channel receiving head/neck coil; T2‐weighted EPI sequence with 1700 ms repetition time, 24 ms echo time, flip angle 70°, each volume 33 × 4 mm slices, matrix size 202 × 202 mm, in plane resolution 3 × 3 mm) at the Advanced Magnetic Imaging Centre of the Aalto University. Anatomical T1‐weighted structural images were acquired with 1 × 1 × 1 mm resolution (MPRAGE pulse sequence, TR 2,530 ms, TE 3.3 ms, TI 1,100 ms, flip angle 7°, 256 × 256 matrix, and 176 sagittal slices).

After the fMRI session, the subjects were presented the narrative again in writing, divided into 128 consecutive coherent phrases (3–5 s in duration), and were instructed to try to recall their interpretation (“what came to your mind”) during listening to the narrative when they first heard it in the scanner and to list, within 20–30 s, words best describing what had come to their minds. There were no limitations as to the type of words (e.g., verbs, substantives, and adjectives) or the amount of words, other than the time limit per segment.

### 2.3 Data analysis

#### 2.3.1 Behavioral data

First, the Finnish conjunctions were removed from the words and all words were translated into English. We then utilized LSA (Landauer & Dutnais, 1997) (implemented using Gensim Python library (Rehurek & Sojka, 2010)) combined with the WordNet knowledge on the content words (Miller, 1995) to estimate similarity/dissimilarity between first three words listed by each subject pair for each 3–5 s segment. English Wordnet was used as there is some loss in lexical variety in the FinnishWordNet (LSA assumes that words that occur in the same context have similar meanings). We used European Parliamentary corpus database (Koehn, 2005) to produce a word co‐occurrence statistic which was turned into a 300‐dimensional (Bradford, 2008) semantic space through singular value decomposition (SVD). Each word list produced by the subjects was represented as a vector in this semantic space and the similarity between word lists was computed as the cosine similarity of the vectors. This LSA‐derived similarity was increased using WordNet knowledge. More specifically, the similarity between words was increased if any of the following relations held.

1. The words were synonyms (e.g., car and automobile).
2. One word was the direct hypernym of the other (e.g., boy and male).
3. One word was the two‐link indirect hypernym of the other (e.g., boy and person).
4. One adjective had a direct similar‐to relation with the other (handsome and beautiful).
5. One adjective had a two‐link indirect similar‐to relation with the other (e.g., handsome and picturesque).
6. One word was a derivationally related form of the other (e.g., man and manly).
7. The words had the same stem but belonged to different parts of speech (e.g., attractive and attraction).

Path distance of one was assigned to category 1, path distance of two to categories 2, 4, 6, and 7, and path distance of three to categories 3 and 5. The new similarity measure between word x and y was derived with the equation

(1) sim(x,y)=simLSA(x,y)+0.5e-αD(x,y)

where *D(x,y)* is the path distance between *x* and *y*. The parameter α was set to 0.25 following previous recommendations (Han, Kashyap, Finin, Mayfield, & Weese, 2013). In case *sim(x,y)* exceeded one, the excess was simply cut and the value set to one. The similarity measure between subjects was obtained by first calculating the similarity in each of the 128 segments by taking the average of the similarity values of all (3*3 = 9) word pairs, and then taking the average of these segment‐wise similarity values.

### 2.4 FMRI data

#### 2.4.1 Preprocessing

The fMRI data were preprocessed with FSL software (www.fmrib.ox.ac.uk/fsl) using the BRAMILA parallel preprocessing pipeline (https://version.aalto.fi/gitlab/BML/bramila). First, after correcting for slice‐timing during acquisition, the EPI volumes were spatially realigned to the middle scan by rigid body transformations to correct for head movements using FSL MCFLIRT. EPI and structural images were coregistered and normalized to each individual's anatomical scan (linear transformation with 9 degrees of freedom with FSL FLIRT; structural images were cleared from non‐brain tissues with FSL BET) followed by a linear transformation from anatomical to standard MNI template space (12 degrees of freedom; FSL FLIRT). Finally, BOLD time series were detrended (linear detrend), motion parameters were regressed out (24 parameters expansion, Power et al. 2014), as well as average signals at deep white matter, ventricles, and cerebrospinal fluid (Power et al., 2014). Finally, a temporal high‐pass filter with a cut‐off frequency of 0.01Hz was applied, followed by spatial smoothing with a Gaussian kernel of 8‐mm FWHM.

The data were analyzed with voxel‐wise comparison of the BOLD signal time courses, by estimating the similarity of the time series using intersubject correlation (ISC, Hasson et al., 2004), examining the temporal similarity of the signals in individual voxels during listening the narrative (Hasson, Malach, & Heeger, 2010; Kauppi et al., 2010; Pajula, Kauppi, & Tohka, 2012). Intersubject correlation was calculated using the ISCtoolbox (Kauppi et al., 2010). We controlled the possible effect of silent pauses (see the effect of stimulus structure on ISC, Lu, Hung, Wen, Marussich, & Liu 2016) by modelling the stimulus structure based on the presence of speech as in Lahnakoski et al. (2012). First, ISC matrices were obtained for each brain voxel by calculating all pairwise Pearson's correlation coefficients (r) of the voxel time courses across the subjects, resulting in 120 unique pairwise r‐values.

To reveal the brain areas related to semantic similarity, we predicted the ISC during listening against the semantic similarity (LSA combined with Wordnet). The significance was tested by conducting a representational similarity analysis using the Mantel test (Mantel, 1967; Nummenmaa et al., 2012). For each voxel, the pairwise BOLD similarity between two subjects in the listening condition was compared to a pairwise semantic similarity score based on the LSA boosted WordNet using Spearman correlation. Since the pairwise similarity values are not independent, a nonparametric approach was used. Surrogate null distribution was approximated with permutations of subject labels for a subset of 101 voxels spanning across the range of the correlation values using kernel density estimation. For each of the 101 voxels, 100,000 permutations were performed. The resulting statistical whole‐brain maps were FWE cluster corrected (cluster‐forming threshold *p* = 0.05, cluster‐extent threshold 125 voxels).

## 3 RESULTS

**Figure 1:**
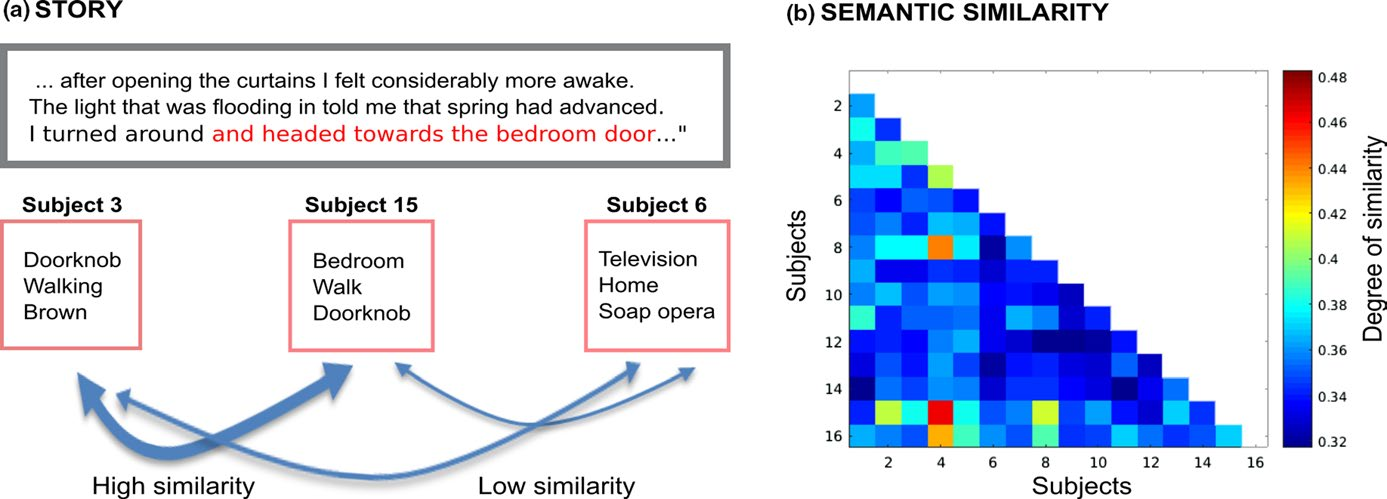
Similarities/differences of subjects' individual semantics when listening to the narrative. LEFT: Excerpt from the narrative with one phrase‐segment highlighted with red font color. Word lists produced by three representative subjects to this particular segment are shown below as examples of similarities and differences in the individual semantics (note that both the narrative excerpt and word lists have been here translated to English for illustration purposes). RIGHT: Correlation matrix showing LSA‐ and WordNet‐derived similarities/differences of subjects' individual semantics when listening the narrative. While some subject pairs exhibit striking similarity, there were also robust differences across many subject pairs. Note that the values plotted here mark mean subject pairwise similarities/differences across the whole narrative

Behavioral responses of the subjects revealed that while some individuals perceived the story semantically similarly (similarity matrix in Figure 1), many subjects differed in how they heard the story as disclosed by LSA (Landauer & Dutnais, 1997) combined with WordNet (Liu et al., 2011; Miller, 1995) knowledge (Han et al., 2013) (Figure 1).

**Figure 2:**
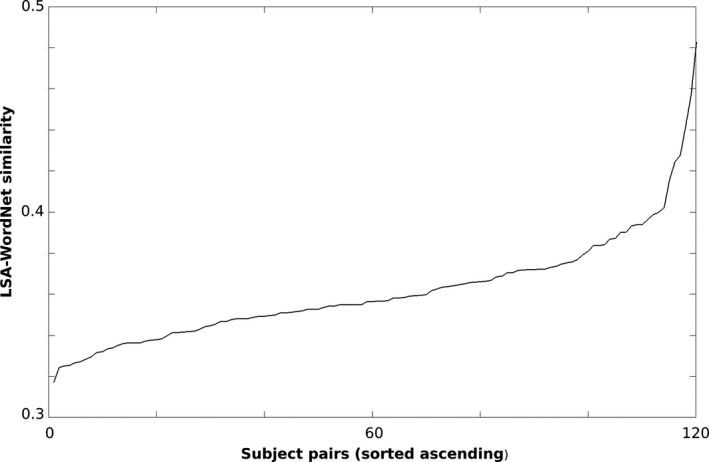
Subjects' pairwise LSA‐Wordnet similarity values (ascending)

A further analysis indicated that the semantic similarities/dissimilarities formed a smooth continuum across subject pairs (Figure 2).

**Figure 3:**
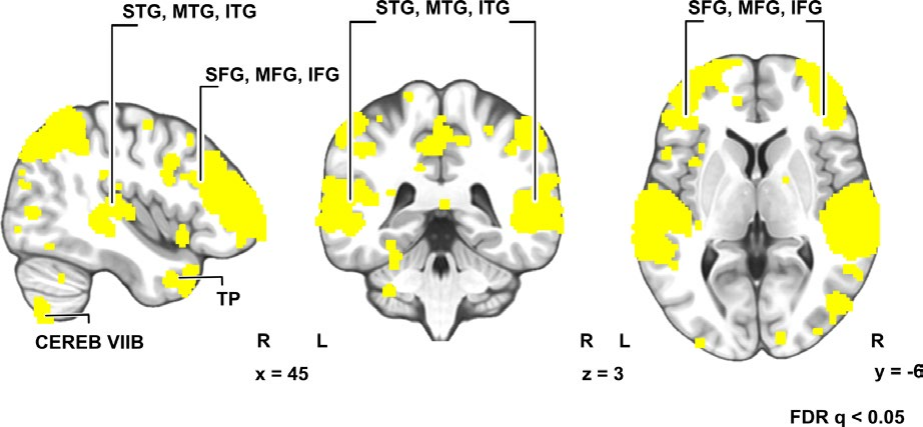
Intersubject correlation (ISC) of BOLD signals (FRD‐corrected q < 0.05)

Intersubject correlation (Hasson et al., 2004; Kauppi et al., 2010) of brain activity during listening the narrative (Figure 3) was statistically significant (FDR‐corrected q < 0.05; across‐all‐voxels mean ISC = 0.0021) in an extensive set of brain areas: bilateral frontal (superior, middle, and inferior frontal gyri), temporoparietal (superior, middle, and inferior temporal gyri) brain areas, extending also to midline regions such as precuneus and cuneus, and right cerebellum. Unthresholded statistical parametric maps of the ISC are available at Neurovault.org//collections/KCKVHDCV/ (Gorgolewski et al., 2015).

**Figure 4:**
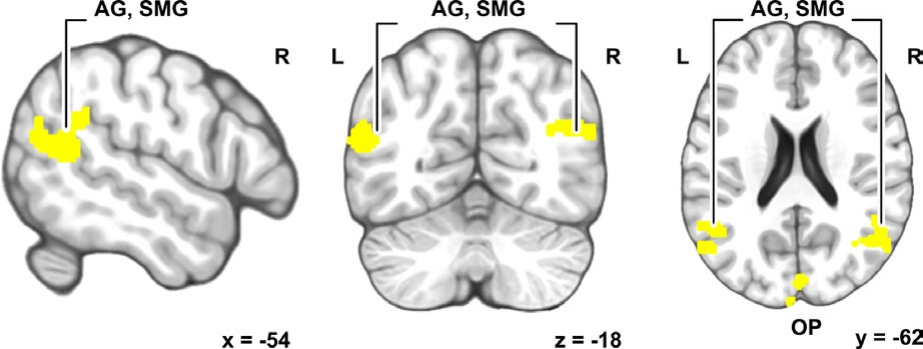
Brain areas where similarities in perceived semantics of the narrative significantly predicted intersubject similarity of brain activity during narrative listening. (AG = angular gyrus; SMG = supramarginal gyrus; OP = occipital pole). Peak activation at left SMG −56, −50, 26, right AG 48, −62, 26, and right cuneus 4, −88, 18. Unthresholded correlation‐value maps from the RSA analysis can be found in 3‐D brain space at Neurovault. org (https://neurovault.org/collections/KCKVHDCV/)

Representational similarity analysis (Kriegeskorte et al., 2008) showed that between‐subject similarities in perceived semantics of the story predicted between‐subject similarities in local brain hemodynamic activity. Subject pairs whose individual semantics were similar also exhibited similar brain activity in bilateral supramarginal and angular gyrus (SMG and AG) of the inferior parietal lobe, and in the occipital pole (Figure 4). Unthresholded statistical parametric maps of the RSA are available at Neurovault.org//collections/KCKVHDCV/ (Gorgolewski et al., 2015).

**Figure 5:**
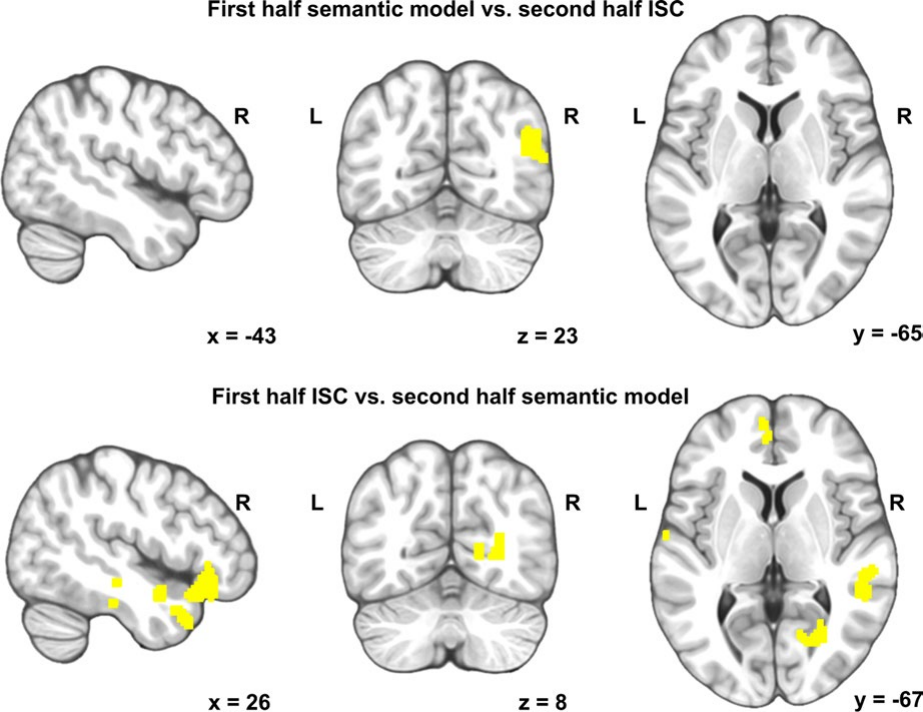
Brain areas where similarities in perceived semantics of the other half predicted ISC of brain activity from the other half. TOP: the first half was used to calculate the similarity of associations (LSA combined with WordNet) and the second half to calculate ISC. BELOW: The first half was used to calculate the ISC and the second half to calculate the similarity of associations (LSA combined with WordNet)

As a control analysis, a Mantel test was performed for split data. The first half was used to calculate the similarity of associations (LSA combined with WordNet) and the second half to calculate ISC (top, Figure 5). The first half was used to calculate the ISC and the second half to calculate the similarity of associations (LSA combined with WordNet) (below in Figure 5). Subject pairs whose individual semantics were similar in the first half of the story, also exhibited similar brain activity in the second half of the story in the AG. Subject pairs whose individual semantics were similar in the second half of the story, exhibited similar brain activity in the first half of the story in scattered clusters in the anterior temporal and frontal areas (Figure 5).

## 4 DISCUSSION

When listening to a captivating story, we often can virtually see the beautiful scenes, various objects, and protagonists acting in their environment (Jacobs, 2015). Such immersion in the flow of a story is a unique human ability made possible by the brain seamlessly calling upon one's own past experiences and acquired generic knowledge to give rise to the vivid mental contents in the form of associations (Bar, 2007) and associated mental imagery (Sadoski et al., 1990). In the present study, we estimated this by asking subjects to list words best describing what had come to their minds as they listened to the narrative during fMRI. Not surprisingly, the subjects were often quite consistent in the word lists they produced, suggesting similarity in their triggered mental experiences. However, word lists from some pairs of subjects were more similar than those of others, suggesting also the presence of individual differences in the propositional meanings and mental imagery elicited by the narrative. While previous studies have shown interindividual differences in, for example, associations elicited during viewing of pictures (Bar, 2007), we present here, to our knowledge novel, methodology to measure and analyze differences in semantics and associated mental imagery elicited by a narrative. For a recent implementation of similar type of approach, see (Nguyen et al., 2019).

Listening to the narrative elicited significant ISC in extensive set of brain areas bilaterally (Figure 2). Similarity of activation extended beyond the classical linguistic areas to bilateral frontal and temporoparietal brain areas, extending to midline regions such as precuneus and cuneus and right cerebellum. Our results are highly similar to those in previous studies using naturalistic linguistic stimuli (Regev, Honey, Simony, & Hasson, 2013; Rowland, Hartley, & Wiggins, 2018; Wilson, Molnar‐Szakacs, & Iacoboni, 2008; Yeshurun et al., 2017). However, significant ISC does not per se reveal brain regions supporting semantics and associated mental imagery elicited by the narrative as significant ISC can be due to similarity in a variety of other cognitive and processes that take place during narrative listening.

Notably, between‐subject similarities in perceived semantics of the story predicted between‐subject similarities in local brain hemodynamic activity in the inferior parietal lobule (SMG and AG) as well as in cuneus in the visual cortex. The SMG and AG belong to the semantic network laid out in a previous meta‐analysis of the semantic system of human brain (Binder et al., 2009) and, supporting recent observations about semantic representations in both left and right hemispheres (Huth et al., 2016), semantic‐related similarity was bilateral. It has been suggested that areas in the inferior parietal lobe function as convergence zones for concepts and event knowledge, and that they receive input from sensory, action, and emotion systems (Binder & Desai, 2011). However, the SMG is also activated by complex motor sequences such as articulation (Oberhuber et al., 2016), and phonological processing (Hartwigsen, Baumgaertner, Price, Koehnke, & Ulmer, 2010), and the activity of SMG has been identified in conditions that pose specific challenge for semantic processing (Price, 2010). Instead, the AG has been shown to be involved in both semantic processing (Binder et al., 2009; Price, 2010) and autobiographical memory, which, in fact, has been suggested to build on general semantic memory processing. Importantly, the AG has been found to serve as a hub in integrating semantic information into coherent representations (Buuren et al., 2015; Price, Peelle, Bonner, Grossman, & Hamilton, 2016), and structural differences in the area have been found to be related to interindividual differences in a task that requires combining of concepts (Price, Bonner, Peelle, & Grossman, 2015). Moreover, given that the heteromodal AG has been indicated to take part in a variety of cognitive functions (Chai, Mattar, Blank, Fedorenko, & Bassett, 2018; Seghier, 2013), the involvement of AG in building individualized semantics and integrating visual processes is plausible.

Similarity of associations predicted similarity of brain activity also in early visual areas (Figure 3), a finding that is in line with previous research suggesting that visual imagery is supported by same areas as visual perception. Results of the current study, therefore, suggest that the narrative may have elicited similar mental imagery for individuals using semantically more similar words to describe what came to their minds during listening of the narrative (Pearson & Kosslyn, 2015). This would not, of course, necessarily imply identical mental images, but rather similarity in the process in which the individuals engaged in generation of the mental imagery during listening to a story. Thus, one can speculate whether individuals with more similar activity in early visual areas drew upon visual information stored in the brain related to objects, scenes, and events in the narrative in similar accuracy or strength (Bergmann et al., 2016).

The practical limitation of our method is that it is highly laborious for experimental subjects to report associations once every 3–5 s for narratives longer than the eight minute one used in the present study. Given this, it is also possible that we might have been able to observe significant activity in some other areas of the semantic network in the present study had we been able to collect more data. Thus, while it can be safely concluded that the inferior parietal and visual cortical areas are involved in generation of individualized semantics and associated mental imagery, one should exercise caution against concluding that some other areas would not be involved in this process. For further inspection, we provide unthresholded statistical parametric maps of the main analysis in Neurovault. For example, when relaxing the statistical threshold, effects are observed in areas such as dorsolateral prefrontal cortex (DLPFC) that have been previously associated with semantic processing at the narrative level (Nguyen et al., 2019). Specifically, Nguyen et al. (2019) collected free recalls from subjects (*N* = 57) after presenting a 7‐min narrative via two different modalities: an animated film without spoken dialogue and an audio description of the animation. By using LSA, they compared across subjects semantic similarity of free recalls of the animation and of the audio description, and observed that greater semantic similarity between subject pairs in their interpretations of the narrative, largely irrespective of modality, predicted ISC in the primary visual areas, premotor cortex, right AG, left SMG, and bilateral superior frontal gyrus. This approach, together with the present one, show that it is possible to quantify interindividual differences of semantic representations and mental imagery during narrative listening in the human brain. Our control analysis also suggests that a subject pair's tendency to elicit similar associations to segments during the first half of the narrative correlates with the pair's tendency to elicit associations to other segments of the story (Figure 5). In future studies, a dynamic analysis looking into neural response corresponding to shorter segments (i.e., phrases or paragraphs) could reveal more detailed information. Notably, RSA analysis could be also optimized (Oswal, Cox, Lambon‐Ralph, Rogers, & Nowak, 2016; Xing, Jordan, & Russell, 2003) to investigate, if the effects in different brain areas are explained by, for example, individuals producing high‐imageability words would be related to more similar activation in the cuneus, and individuals who are more similar in high‐level semantics would exhibit higher similarity in the AG.

The method introduced in the current paper could be potentially applied in a number of settings where self‐report methods are needed, alone and in combination of other (e.g., neuroimaging) measures, to estimate the “mental contents” of experimental subjects. This could include, for example, usability research where the recording of testing a human–machine interface is played back to the test subjects afterwards and they are asked to produce word lists describing what was on their minds during the testing. Clinical research might also benefit from the method, as it is possible to assess the thought patterns of patients compared to healthy volunteers while they, for example, watch a movie containing social interactions during neuroimaging, as significant differences in brain activity have been observed between, for example, high‐functioning autistic and neurotypical subjects, yet specific behavioural measures of differences in interpretation have been lacking (Glerean et al., 2016).

In conclusion, individuals with more similar activity in the SMG and AG of the inferior parietal lobe, as well as in early visual cortical areas, specifically cuneus, during listening to a narrative also elicited mental associations that were semantically more similar. During listening to a captivating narrative, the inferior parietal lobe and early visual cortical areas seem, thus, to support elicitation of individual meanings and flow of mental imagery.

## CONFLICT OF INTEREST

None of the authors claims any conflict of interest. None of the authors reports any competing financial interests.

## AUTHOR CONTRIBUTIONS

SS planned the experiment, prepared the stimuli, collected and analyzed the data, prepared the figures, and wrote the manuscript as first author. JA planned the experiment, collected and analyzed the data, prepared the figures, and wrote the manuscript. EG analyzed the data, prepared the figures, and wrote the manuscript. MK analyzed the data. TH planned the experiment and supervised the data analysis. MS planned the experiment, supervised the collecting data, and wrote the manuscript. MB planned the experiment, supervised the data analysis, and wrote the manuscript. IPJ planned the experiment, supervised the data analysis, prepared the figures, and wrote the manuscript.

## DATA AND MATERIALS AVAILABILITY

Unthresholded correlation‐value maps from the RSA analysis can be found in 3‐D brain space at Neurovault.org//collections/KCKVHDCV/ (Gorgolewski, et al., 2015).
