# Supplementary material for: Inferior parietal lobule and early visual areas support elicitation of individualized meanings during narrative listening
Source: Brain Behav. 2019 Apr 11;9(5):e01288. doi: 10.1002/brb3.1288 (PMC6520291; doi:10.1002/brb3.1288)
Supplement: Supplementary file 1 [file BRB3-9-e01288-s001.docx]

Supplementary Materials

Appendix SA. Stimulus narrative in Finnish

Havahduin herätyskellon sinnikkääseen pirinään. Herääminen tuntui raskaalta, mutta pakotin itseni ylös sängystä. Sammutin ilkeää ääntä pitäneen herätyskellon ja venyttelin hetken. Puolinukuksissa petasin vuoteeni ja vedin puseron ja housut päälleni. Avattuani verhot piristyin huomattavasti. Valon tulvahdus kertoi siitä että kevät oli jo pitkällä. Käännyin ympäri ja suuntasin kohti makuuhuoneen ovea. Matkalla jalkani osui sängyn takana lojuneeseen löylyämpäriin joka rämisi kaatuessaan. Piti oikein sadatella hetki, kun kipu vihloi varvasta, mutta sitten nostin ämpärin pystyyn ja suuntasin kohti keittiötä.

Menin suoraan jääkaapille. Purkki jogurttia ja päärynä olivat riittävä aamupala. Jääkaapissa oli jostain syystä myös mieheni Jarkon matkapuhelin. Alkoi olla jo kiire lähteä töihin, mutta keitin kuitenkin kahvia ja sen tuoksu leijui ihanasti sieraimiini. Yhtäkkiä tunsin käsien alkavan hieroa hartioitani. Jarkko oli hiipinyt taakseni ja pääsi yllättämään minut iloisesti.Kun ihmettelin, miksi Jarkko ei vielä ollutkaan töissä, hän vastasi tunteneensa aamuyöstä olonsa pahoinvoivaksi. Hän aikoi mennä kohta takaisin lepäämään.

Aamiaiskahvini loppuja vielä hörpätessäni annoin taudista uupuneelle Jarkolle eteisessä lähtösuukon. Vedin takin harteilleni, laitoin kengät jalkaani ja astahdin ulos. Kaunis linnunlaulu täytti ilman ulko-oven auetessa. Sora ratisi kenkien alla, kun kiirehdin autolle. Ennen ratin taakse hyppäämistä huomasin onneksi Jarkon repun katolla. Niin tyypillistä Jarkkoa, hymähdin mielessäni, kun kannoin reppua sisään. Kun laskin repun eteisen lattialle, huomasin Jarkon nopeasti lopettavan puhelun ja punastuvan melkein kuin syyllisen näköisenä. Jarkko kuitenkin vakuutti että joku oli vain soittanut väärään numeroon. Minulla oli jo kiire joten en kysellyt enempiä vaan kiirehdin autolle.

Moottori hyrräsi tasaisesti, kun ajoin rauhaisan maaseutumaiseman läpi. Maasto kumpuili metsien ja peltojen vaihdellessa. Harjanteilla kasvoi ilmavaa mäntymetsää ja notkelmissa tiheää kuusikkoa. Muutamissa kohdin tie ylitti alla kohisevia pieniä jokia. Luonto alkoi jo vihertää kevätauringon herättävästä vaikutuksesta.

Pohdin Jarkon aamuista käytöstä: puhelun äkillistä katkaisua ja punastumista ikään kuin syyllisyyden tunnoissa. Mietin, onkohan Jarkolla peliä jonkun kanssa. Entä aavistaakohan hän että epäilykseni heräsivät ja, jos aavistaa niin, pelästyyköhän ja lopettaa suhteen. Uskaltaisinko sopivan tilaisuuden tullen vakoilla Jarkon puhelimesta kuka soittaja oli ollut? Epäilisiköhän Jarkko moista yritystä ja olisiko hän jo tyhjentänyt puhelutiedot? Ehkä minun pitäisi kovistella Jarkkoa jos puhelutiedot onkin tyhjennetty.

Havahduin näistä synkistä mietteistä todellisuuteen, kun iso puu kaatui ryskyen tien poikki. Jarrut kirskuivat ja sain vaivoin auton pysäytettyä ennen törmäystä! Nousin ulos nähdäkseni mikä puun oli kaatanut. Yllätyksekseni näin ruskeakarvaisen talttahampaisen otuksen puun juurella, ja rungossa oli vahvat jyrsintäjäljet. Majava! Lähestyessäni sitä se pakeni syvemmälle metsään. Vaikka olimme asuneet jo viitisen vuotta Kanadassa, tämä oli ensimmäinen kerta kun näin majavan. Pudistelin päätäni, kiirehdin autolle, käänsin auton ympäri ja suunnittelin mielessäni vaihtoehtoisen reitin töihin. Samalla otin puhelimen taskustani ja soitin paikalliseen hätänumeroon ilmoittaakseni tielle kaatuneesta puusta.

Astuessani hissiin työpaikan parkkihallista törmäsin Jamesiin, komeaan mieheen jonka kanssa minulla oli ollut salaromanssi syksyllä. James oli kuitenkin ruvennut epäilemään että hänen vaimonsa aavistaa jotain ja niin sovimme, että pidämme taukoa ja annamme pölyn laskeutua. Epäilin tosin, että James oli vain kyllästynyt minuun ja että hänen vaimonsa ei oikeasti epäillyt mitään. Voihan olla, että Jamesilla oli joku toinen rakastaja, eikä hän ollut halunnut sekoittaa liiaksi pakkaa. Mutta salasin epäilykseni ja juttelin niitä näitä. Olimme Jamesin kanssa menossa samaan kokoukseen. Matkalla kokoushuoneeseen huomioni kiinnitti moottorisaha, jonka joku oli jättänyt käytävälle.

Kokous osoittautui tiukaksi paikaksi. Tarkoitus oli sopia tavaran toimittamisesta mutta epäilin heti alusta lähtien toisen osapuolen kykyä toimittaa sillä aikataululla ja hinnalla kuin mitä esittivät. Kokouksen kuluessa myyntimiehet alkoivat selvästi hermostua tajutessaan, että en ollut valmis nielemään heidän tarinoitaan. Lopulta päätettiin vain että palaamme asiaan yön yli mietittyämme.

Kokoushuoneesta ulos astuessani minua odotti yllätys. Työpaikan väki oli kokoontunut kahvihuoneeseen ja he onnittelivat minua aurinkoisesti viiden vuoden työrupeamasta. Samalla minulle annettiin lahjaksi moottorisaha! Se samainen, jota ihmettelin käytävällä. Toki porukat töissä tiesivät että asuin maalla ja siellä sahalle olisi käyttöä. Samalla epäilin kyllä, että taustalla piili vähän sarkasmiakin. Olinhan toimitusjohtajuuteni aikana leikannut useammankin vähemmän tuottoisan rönsyn firmasta pois.

Appendix B Stimulus narrative translated to English.

I woke up to the persistent sound of my alarm clock. Waking up felt heavy, but I forced myself out of bed. I turned off the nasty sound of my alarm clock and stretched for a moment. Half asleep I made my bed and pulled a blouse and trousers on me. After opening the curtains, I felt considerably more awake. Light that was rushing in told me that spring had advanced. I turned around and headed towards the bedroom door. On my way, my foot hit a sauna bucket which was lying behind the bed and made a ruckus when falling. I had to curse for a moment when the pain hit my toes, but then I lifted the bucket up and headed towards the kitchen.

I went straight to the fridge. Jar of yoghurt and pear were an adequate breakfast. For some reason, my husband Jarkko’s mobile phone was also in the refrigerator. I was beginning to be in a hurry for work, but I nonetheless brewed some coffee, the aroma of which floated delightfully into my nose. Suddenly I felt hands begin to rub my shoulders. Jarkko had appeared behind me and he managed to surprise me pleasantly. When I wondered why Jarkko had not yet left for work, he replied that he had felt nauseous during the small hours. He was going to go right back to rest.

While sipping the last of my breakfast coffee, I gave the weary Jarkko a kiss. I pulled a jacket on me, I put my shoes on and stepped outside. As the door opened, beautiful birdsong filled the air. Gravel rattled underneath my shoes as I hurried to the car. Before jumping behind the wheel, I noticed Jarkko's backpack on the roof of my car. So typical, I smiled to myself, as I carried the backpack back inside. When I left it on the floor in the hallway, I noticed Jarkko quickly stopping a phone call and blushing almost as if guilty. However, Jarkko assured that someone had just called the wrong number. I was in a hurry so I asked no further questions, but hasted to my car.

Engine ran steadily, as I drove through the peaceful rural landscape. The terrain varied with forests and fields. On ridges grew pines and in valleys dense spruce. In other places the road crossed over small rapids. Nature already started to turn green, much to the influence of the spring sun.

I reflected on the behaviour of Jarkko this morning: his sudden disconnection of the phone call and blushing as if guilty. I wonder whether Jarkko had something inappropriate going on with someone. Would he guess that he had awakened my doubts, and, if so, would he be scared enough to terminate the relationship. I wondered if I would dare to spy from Jarkko’s phone who the caller was if I had the opportunity? Would Jarkko suspect that and empty his phone-call records? Maybe I should confront Jarkko if the call data had been cleared.

I woke to the reality from these gloomy reflections, when a large tree fell with a crash across the road. Brakes screeched as I struggled to stop the car before a crash! I climbed out of the car to see what had downed the tree. To my surprise, I saw a brown-haired, square teethed creature at the foot of the tree, and the root of the tree had bite marks. Beaver! When I approached the beaver, he fled deeper into the forest. Although we had already lived for about five years in Canada, this was the first time I saw a beaver. Shaking my head, I hastened back to the car, I turned the car around and I planned an alternate route to work in my mind. At the same time I took the phone from my pocket and I called the local emergency number, to declare fallen timber.

As I entered the elevator in the parking garage of my working place, I came across James, a handsome man with whom I had had a secret romance in the fall. James had begun to suspect that his wife knows something, and then we agreed that we take a break and let things cool down. I suspected, however, that James was just tired of me, and that his wife had actually not suspected anything. It may well be that James had another lover, and he did not want to mix things up too much. But I hid my doubts and I chit chatted with him. James and I were going to the same meeting. On the way to a meeting room, a chain-saw that someone had left in the corridor caught my attention.

The meeting proved to be a tight match. The aim was to agree on delivery of some goods, but I doubted from the very beginning the other party's ability to deliver on the schedule and at the price required. During the meeting, the salesmen began to get nervous, when they realized that I was not ready to swallow their excuses so easily. In the end, it was only decided that we will get back to the matter after giving it some thought overnight.

There was a surprise waiting for me when I stepped out of the office. A crowd of colleagues had gathered in the coffee room and they congratulated me for my five-year time tenure at the firm. I was given a chainsaw as a gift! The very same one, which I had wondered about in the corridor. Sure, the people at work knew that I lived in a rural area and a chainsaw would be of use. At the same time, I doubted, though, that there was a bit of sarcasm included. During my time as a CEO I had cut more than one less lucrative branch of the company off.
